# Extracellular volume fraction provides more imformation than fibrosis

**DOI:** 10.1186/1532-429X-17-S1-Q134

**Published:** 2015-02-03

**Authors:** Shi-Jun Zhang, Shenghong Ju

**Affiliations:** 1 Department of Radiology, Zhongda Hospital, Southeast University, Nanjing, China

## Background

Extracellular volume fraction (ECV) has been applied to the detection of diffuse myocardial interstitial fibrosis or exudative pathologic changes in various clinical settings, but there is lack of study to date concerning the significance of ECV at the early phase of left ventricular remodeling. We intended to assess myocardial ECV in essential hypertension and to study the implications of ECV to the early phase of left ventricular remodeling.

## Methods

Thirty-six healthy volunteers and 39 essential hypertensive patients underwent cardiac magnetic resonance imaging to study left ventricular morphological and functional measures and ECV. Normalized intracellular volume (nICV) and normalized extracellular volume (nECV) were also calculated based on ECV and normalized left ventricular myocardial volume. Independent samples *t* test and Pearson correlation analysis were chiefly used in statistical analysis.

## Results

Women's ECV was higher than men's in normotensive subjects (p < 0.001). In women, ECV of hypentensive subjects was lower than that of normotensive subjects (p = 0.028). ECV correlated with nICV (*r* = 0.645, p < 0.001) but not with nECV (p = 0.526). nICV correlated with left ventricular concentricity (*r* = 0.741, p < 0.001), E/A (*r* = -0.542, p < 0.001), and Time To Peak Filling (*r* = 0.313, p = 0.006). While nECV only correlated with left ventricular concentricity (*r* = 0.488, p < 0.001) and E/A (*r* = -0.287, p < 0.013) with smaller Peason correlation coefficients.

## Conclusions

Women have higher myocardial ECV than men. In normal subjects and hypertensive patients at an early stage of left ventricular remodeling, nICV is the dominant factor to directly influence ECV, and has closer ties than nECV with left ventricular concentric remodeling and diastolic function. The ECV should be prudently used as a fibrosis index especially in the context of ventricular remodeling involving remarkable cardiac myocyte hypertrophy.

## Funding

This work was supported by Major State Basic Research Development Program of China (973 Program, No. 2013CB733802) and National Nature Science Foundation of China (NSFC, No. 81371538).

